# A dual-branch multidimensional attention fusion network for EEG-based fatigue classification

**DOI:** 10.3389/fphys.2026.1923970

**Published:** 2026-09-09

**Authors:** Jingqing Lu, Shihong Liu, Wei Li, Chenyu Mu, Diankun Gong, Dezhong Yao

**Affiliations:** 1 The Clinical Hospital of Chengdu Brain Science Institute, MOE-K Lab for NeuroInformation, Brain-Apparatus Communication Institute, University of Electronic Science and Technology of China, Chengdu, China; 2 Sichuan Institute for Brain Science and Brain-Inspired Intelligence, University of Electronic Science and Technology of China, Chengdu, China; 3 School of Information Engineering, Nanchang Hangkong University, Nanchang, China

**Keywords:** attention mechanism, deep learning, electroencephalography, fatigue classification, spatial-temporal features

## Abstract

**Introduction:**

Electroencephalogram (EEG)-based fatigue classification is important for vigilance monitoring. Reliable recognition remains challenging due to fatigue-related neural changes that involve both spectral-temporal dynamics and altered inter-channel interactions.

**Methods:**

This study develops a lightweight Dual-Branch Multidimensional Attention Fusion Network (DB-MDAFNet) that integrates spectral-temporal features and PCC-derived inter-channel dependency representations for EEG-based fatigue classification. The framework uses differential entropy features extracted from five canonical EEG frequency bands as input. A parameter-free multidimensional enhancement (PF-ME) module is applied for feature recalibration. It then extracts complementary representations via a Channel Attention-Multi-scale Temporal (CA-MT) branch and a Functional Connectivity-Topological Encoding (FC-TE) branch derived from Pearson correlation coefficients (PCC). The fused representation is used to classify EEG samples into alert, tired, and drowsy states. Experiments were conducted on the public SEED-VIG dataset and the self-constructed video game-based fatigue dataset (DVG).

**Results:**

Under identical subject-dependent settings, DB-MDAFNet achieved 85.58% accuracy on SEED-VIG and 97.12%–98.56% accuracy on DVG datasets. The model maintained a compact architecture, with 0.185 M parameters under the DVG input configuration. Ablation results indicated the complementary contribution of the two branches. Model interpretability analyses suggested that the learned representations contained spatially structured patterns across frontal, parietal, and occipital electrode regions.

**Discussion:**

The proposed framework, integrating multi-scale temporal learning with connectivity-derived topological encoding, provides a lightweight and interpretable architecture for EEG-based fatigue monitoring.

## 1 Introduction

Fatigue is a major factor affecting human performance and safety in transportation, aviation, and industrial work environments (Phillips et al., 2017; Bendak and Rashid, 2020). Prolonged cognitive demands and monotonous tasks can reduce attention, slow reaction times, and impair decision-making. Reliable fatigue monitoring is therefore important for safety management, human-machine interaction, and health informatics. Fatigue assessment generally relies on subjective reports or objective physiological and behavioral measurements (LaChapelle and Finlayson, 1998; Shahid et al., 2010; Díaz-García et al., 2022). Among physiological measures, scalp EEG non-invasively records electrical activity with millisecond-scale temporal resolution (Biasiucci et al., 2019). Accordingly, EEG has been widely used to characterize affective states (Li et al., 2022), support neurorehabilitation (Leamy et al., 2014; Ang and Guan, 2017; McDermott et al., 2023), and monitor fatigue in safety-critical environments (Chai et al., 2017; Subasi et al., 2022; Xu et al., 2023).

Neurophysiological evidence indicates that fatigue and cognitive load are associated with frequency-specific changes in EEG activity (Klimesch, 1999; Antonenko et al., 2010; Borghini et al., 2014). Theta and alpha activity generally increase as fatigue develops (Goodman et al., 2025; Liu et al., 2025), whereas beta activity tends to decrease with reduced alertness (Li et al., 2020; Tran et al., 2020; Matta et al., 2025). Fatigue is also associated with alterations in large-scale functional networks, including the default mode network (Gergelyfi et al., 2021). Together, these findings motivate EEG representations that retain both spectral-temporal activity and inter-channel relationships. However, EEG-based fatigue classification remains difficult because EEG signals are nonstationary, noise-sensitive, and highly variable across individuals (Luo et al., 2024; Rudroff, 2026).

Deep learning (LeCun et al., 2015) methods, including convolutional neural networks (CNNs) (Rajwal and Aggarwal, 2023), recurrent neural networks (RNNs) (Güler et al., 2005), and graph neural networks (GNNs) (Klepl et al., 2024), have improved automatic EEG feature extraction. Subasi et al. (Subasi et al., 2022) combined a flexible analytic wavelet transform with multi-boosting to distinguish fatigue from alertness. Wang et al. (Wang et al., 2024) employed a deep residual shrinkage network to suppress noise and redundant EEG features recorded in a construction environment. To address inter-subject variability, Liu et al. (Liu et al., 2020) investigated transfer learning for cross-subject mental-fatigue recognition. Attention mechanisms (Niu et al., 2021) adaptively reweight feature representations and have been widely incorporated into deep neural networks. Representative approaches include squeeze-and-excitation blocks (Hu et al., 2018) for channel recalibration and transformer self-attention for sequence modeling (Vaswani et al., 2017). Neural processing depends on coordinated activity across distributed brain regions rather than isolated regional responses (Fries, 2005; Uhlhaas and Singer, 2006). Functional brain networks have been shown to reconfigure across cognitive loads (Zhang et al., 2023). Qi et al. (Qi et al., 2020) examined coherence, phase-lag index, and partial-directed coherence-based networks to predict individual behavioral impairment induced by mental fatigue. Chen et al. (Chen et al., 2024) combined PCC and magnitude-squared coherence within a dual-stream GCN to distinguish alert and fatigued states.

Nevertheless, existing EEG fatigue classifiers differ in how they represent temporal dynamics, frequency-band information, and inter-channel dependencies. Moreover, flattening or compressing frequency-band information during feature extraction may obscure band-specific physiological patterns. The limited availability of dedicated EEG fatigue datasets restricts model development and independent evaluation.

To address these limitations, this study constructs an EEG fatigue dataset based on video game (DVG) and proposes a Dual-Branch Multidimensional Attention Fusion Network (DB-MDAFNet). The DVG paradigm combines action-video-game-based fatigue induction with self-reported fatigue assessment and resting-state EEG acquisition. DB-MDAFNet comprises a parameter-free multidimensional enhancement module (PF-ME), a channel-attention multi-scale temporal branch (CA-MT), and a functional-connectivity topological encoding branch (FC-TE). These components jointly capture band-specific temporal dynamics and inter-channel connectivity while limiting additional trainable parameters. The main contributions of this study are as follows:

1. An EEG fatigue dataset (DVG) was constructed using an action video-gaming task to induce mental fatigue, with concurrent collection of self-reported fatigue ratings and EEG data.
2. A dual-branch EEG fatigue classification model, DB-MDAFNet, was designed to combine multi-scale temporal feature learning with Pearson correlation coefficient (PCC)-based functional connectivity representation while preserving the channel, frequency-band, and temporal structure of differential entropy (DE) features.
3. The proposed method was evaluated on both the public SEED-VIG and DVG datasets under a subject-dependent protocol. Comparative tests and ablation analyses evaluated classification performance, and descriptive visualizations illustrated the learned feature weight distributions.

## 2 Materials and methods

### 2.1 Dataset

DB-MDAFNet was evaluated on the self-constructed DVG dataset and the public SEED-VIG dataset.

#### 2.1.1 Self-constructed dataset (DVG)

Fourteen male students (mean age: 23 years) from the University of Electronic Science and Technology of China were recruited. Two participants withdrew for personal reasons, and data from the remaining twelve participants were analyzed. None of the participants reported extensive or long-term video-gaming experience. All participants were right-handed, had normal or corrected-to-normal vision, and reported no history of neurological disorders or substance misuse. Participants were instructed to avoid coffee, tea, alcohol, sleep deprivation, and strenuous exercise before each session. The protocol was approved by the Departmental Ethics Committee of the University of Electronic Science and Technology of China and complied with the Declaration of Helsinki. All participants had written informed consent before participation.

**Figure 1:**
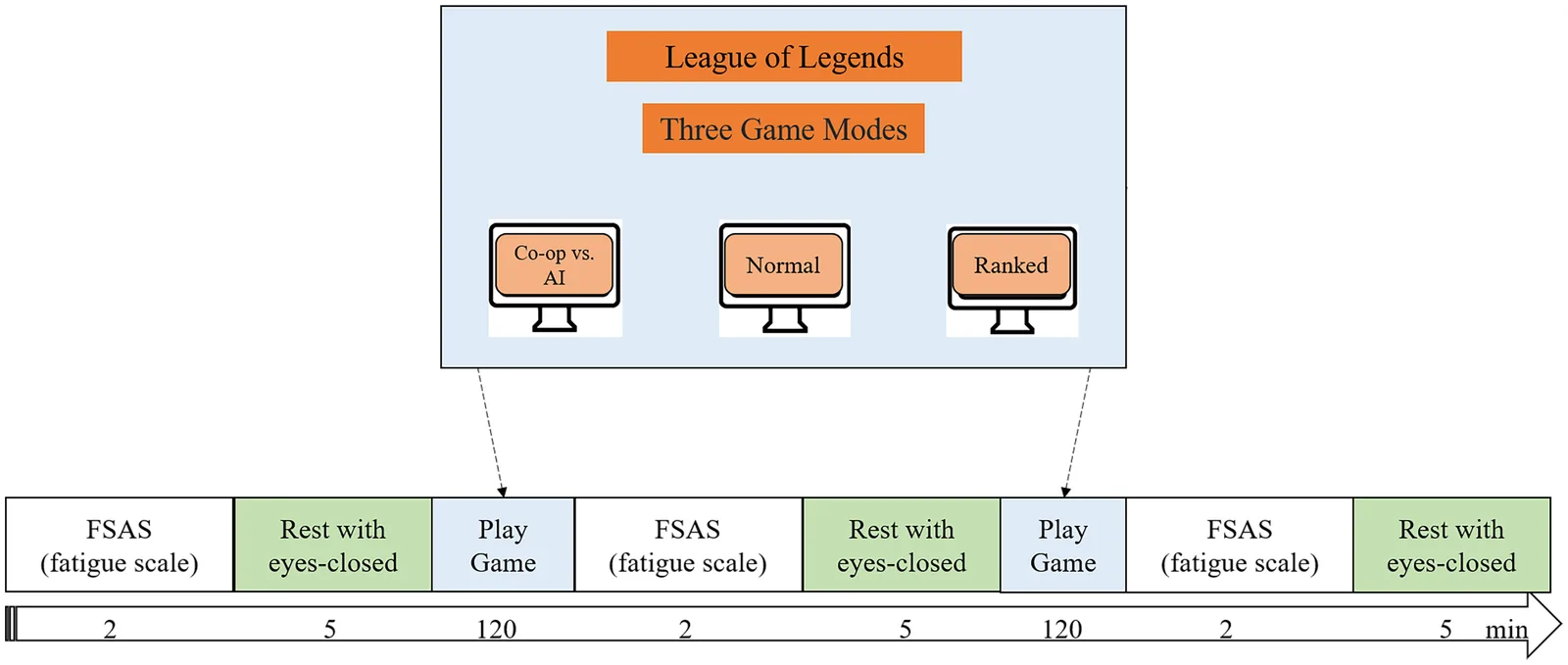
Experimental paradigm of the DVG datasets.

The DVG paradigm (**Figure 1**) combined video-game-based fatigue induction, eyes-closed resting-state EEG recording, and the modified Fatigue Self-Assessment Scale (FSAS) (Wang and Xue, 2009). EEG was recorded using a 32-channel BioSemi Active Two System, with electrodes positioned according to the extended international 10–20 system (Klem et al., 1999). EEG signals were sampled at 2048 Hz. The mental fatigue subscale of the modified FSAS was administered at each EEG recording time point. Each item of the modified FSAS was rated from 0 to 9, with higher scores indicating greater agreement with the described condition.

**Figure 2:**
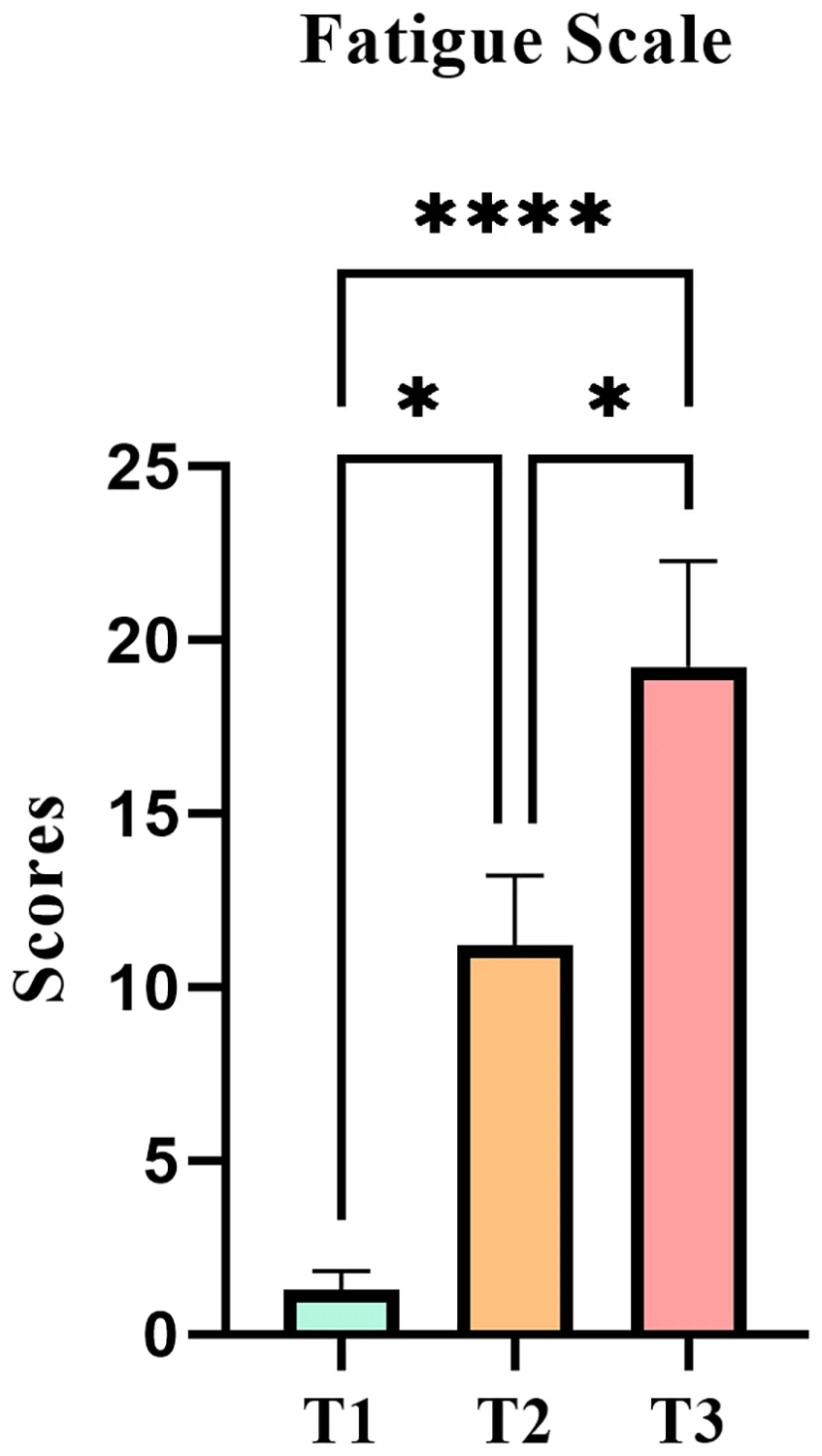
Average FSAS scores at three time points. Error bars represent standard errors of the mean (SEM). *p < 0.05, ****p < 0.0001.

The three DVG datasets corresponded to Co-op vs. AI (DVG1), Normal (DVG2), and Ranked (DVG3) game modes. Each participant completed one mode per session. The session order was counterbalanced. Sessions were separated by at least three days to reduce potential carryover effects. During each 4-h session, the mental fatigue subscale and 5-min eyes-closed EEG recording were obtained at baseline (T1), 2 h (T2), and 4 h (T3). According to the predefined experimental stages, EEG samples collected at T1, T2, and T3 were operationally labeled as alert, tired, and drowsy, respectively. Then, FSAS scores at each time point were used to validate the stage-based labeling for each participant. The session-averaged FSAS scores (**Figure 2**) increased significantly across the three time points (Friedman test: χ² (2) = 24, p < 0.0001). Furthermore, Dunn’s *post hoc* tests showed significant differences between T1 and T2 (adjusted p = 0.0429), T2 and T3 (adjusted p = 0.0429), and T1 and T3 (adjusted p < 0.0001).

#### 2.1.2 Public SEED-VIG dataset

SEED-VIG (Zheng and Lu, 2017) is a public vigilance-estimation dataset collected during simulated driving. It includes 23 healthy adults (11 males, 12 females; age: 23.3 ± 1.4 years) who completed an approximately 2-h monotonous driving task using a virtual-reality driving system. EEG was acquired using a Neuroscan system at 1,000 Hz, and eye movements were recorded synchronously using SMI eye-tracking glasses. Vigilance was annotated using the percentage of eyelid closure (PERCLOS) derived from the eye-tracking recordings (Equation 1):

(1) $$PERCLOS=\frac{blink+CLOS}{blink+CLOS+fixation+saccade}$$

where blink, fixation, saccade, and CLOS denote the durations of blinks, fixations, saccades, and eye closures, respectively. For the present three-class experiments, PERCLOS (P) values of P<0.35, 0.35≤P<0.70, and P≥0.70 were assigned to the alert, tired, and drowsy classes, respectively.

### 2.2 Data preprocessing

Initially, EEG signals were processed with band-pass filtering (0.5–50 Hz) to remove slow baseline drifts and high-frequency noise. In addition, a 50-Hz notch filter was applied to suppress power-line interference. EEG was re-referenced to the infinite reference via REST (Yao, 2001). Ocular, muscle, and cardiac artifacts were removed using independent component analysis (ICA) (Jung et al., 2000). Noisy channels were interpolated when necessary. Subsequently, resampling and segmentation were performed. The signals were down-sampled to 1000 Hz (DVG datasets) and 200 Hz (SEED-VIG dataset), with corresponding epoch lengths of 3.5 s and 8 s, respectively. Epochs were generated using non-overlapping windows.

Multi-band filtering was conducted using a fifth-order Butterworth band-pass filter to decompose the EEG signals into five canonical bands, namely delta (0.5–4 Hz), theta (4–8 Hz), alpha (8–12 Hz), beta (12–30 Hz), and gamma (30–50 Hz). Finally, DE features were calculated for each EEG channel and frequency band by the following formulas (Equations 2, 3):

(2) $$DE=\frac{1}{2}\ln(2\pi e\sigma^{2})$$

(3) $$\sigma^{2}=\frac{1}{m}\sum_{i=1}^{m}{\left(x_{i}-\mu \right)}^{2}$$

where m denotes the number of sampling points within the sub-window, $x_{i}$ is the i-th sample of the EEG signal, and μ and $\sigma^{2}$ are its mean and variance.

### 2.3 Model architecture

**Figure 3:**
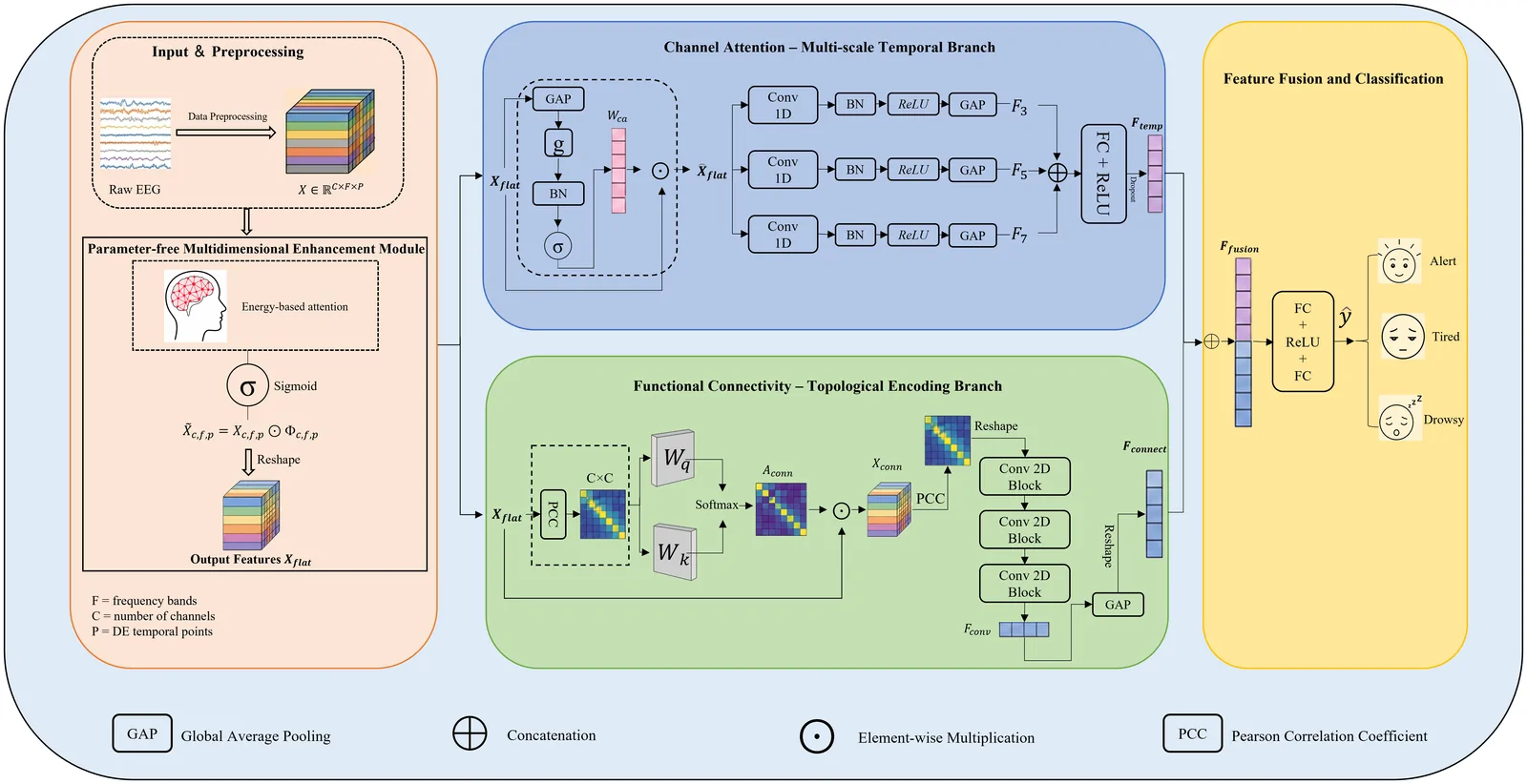
Overall architecture of the DB-MDAFNet.

As shown in **Figure 3**, DB-MDAFNet consists of a shared parameter-free multidimensional enhancement (PF-ME) module, two parallel feature-extraction branches (CA-MT and FC-TE), and a fusion classifier. The input is the DE feature tensor $X\in {\mathbb{R}}^{B\times C\times F\times P}$, where *B, C, F*, and *P* (Equation 2, 3) denote the batch size, number of channels, frequency bands, and DE temporal points, respectively. PF-ME first performs element-wise recalibration without additional trainable parameters. The recalibrated tensor is then passed to two parallel branches. The CA-MT branch combines channel attention with multiscale one-dimensional convolutions to extract channel-time-aware representations from the concatenated band-time sequence. In parallel, the FC-TE branch uses PCC-derived attention and two-dimensional convolutions to encode inter-channel connectivity patterns. The two branch features are concatenated and passed to the classification head. The classifier produces predicted labels for alert, tired, and drowsy states.

#### 2.3.1 Parameter-free multidimensional enhancement module

To enhance the model’s capacity to capture critical features in EEG signals, we incorporate a PF-ME module based on SimAM. For each feature map, the squared deviation of an element from the corresponding mean is (Equation 4):

(4) $${\hat{X}}_{c,f,p}={\left(X_{c,f,p}-\mu_{f}\right)}^{2},\;\mu_{f}=\frac{1}{C\times P}\sum_{c=1}^{C}\sum_{p=1}^{P}X_{c,\;\;f,p}$$

where ${\hat{X}}_{c,f,p}$ denotes the squared difference value for the *c-*th channel at the *p-*th time point in the *f-*th frequency band. $\mu_{f}$ denotes the frequency-wise global mean. The corresponding variance estimate is (Equation 5):

(5) $$\nu_{f}=\frac{1}{C\times P-1}\sum_{c=1}^{C}\sum_{p=1}^{P}{\hat{X}}_{c,f,p}$$

The element-wise weight _*c,f,p*_ is calculated as (Equation 6):

(6) $$c,f,p=\sigma (\frac{{\hat{X}}_{c,f,p}}{4(\nu_{f}+\epsilon )}+\frac{1}{2})$$

where ϵ is a small constant (set to 10^-4^) to ensure numerical stability, and σ(·) is the sigmoid function. The recalibrated tensor ${\tilde{X}}_{c,f,p}$ is obtained through an element-wise product (Equation 7):

(7) $${\tilde{X}}_{c,f,p}=X_{c,f,p}{⊙}_{c,f,p}$$

The enhanced tensor $\tilde{X}$ is reshaped to a channel-centered layout, and the frequency and time dimensions are concatenated in a fixed order (Equation 8):

(8) $$X_{flat}=Reshape(\tilde{X},[B,C,L])$$

where $X_{flat}\in {\mathbb{R}}^{B\times C\times L}$ represents the reshaped alignment matrix, with L=F×P denoting the integrated sequence length. This operation effectively fuses spectral and temporal information into a unified spatial feature space.

#### 2.3.2 Channel attention – multiscale temporal branch

The CA-MT branch learns channel-weighted features from $X_{flat}$. A channel descriptor is obtained by averaging across the sequence dimension (Equation 9):

(9) $$ɡ=\frac{1}{L}\sum_{l=1}^{L}X_{flat}(:,:,l)\in {\mathbb{R}}^{B\times C\times 1}$$

Subsequently, batch normalization and sigmoid activation are used to generate the channel weights (Equation 10):

(10) $$W_{ca}=\sigma (BN(ɡ))\in {\mathbb{R}}^{B\times C\times 1}$$

where $W_{ca}(b,c,1)$ denotes the weight assigned to channel c in sample b. The recalibrated feature ${\hat{X}}_{flat}$ is obtained via an element-wise Hadamard product (Equation 11):

(11) $${\hat{X}}_{flat}=X_{flat}⊙W_{ca}$$

The weighted tensor ${\hat{X}}_{flat}$ is then processed by three parallel Conv1D paths with kernel sizes $k_{i}\in \{3,5,7\}$ (Equation 12):

(12) $$F_{i}=GAP(ReLU(BN(Conv1D_{k_{i}}({\hat{X}}_{flat}))))$$

where GAP(·) denotes global average pooling. The resulting multi-scale feature vectors are concatenated to form a unified representation (Equation 13):

(13) $$F_{multi}=Concat[F_{3},F_{5},F_{7}]$$

Finally, a fully connected layer (FC), ReLU activation, and dropout operation are applied to produce a 128-dimensional feature $F_{temp}\in {\mathbb{R}}^{B\times 128}$ (Equation 14).

(14) $$F_{temp}=Dropout(ReLU(FC(F_{multi})))$$

#### 2.3.3 Functional connectivity – topological encoding branch

FC-TE encodes PCC-based inter-channel connectivity patterns. An initial PCC matrix is computed across the concatenated band-time sequence $X_{flat}$ (Equation 15):

(15) $$M_{PCC(u,v)}=\frac{Cov(X_{flat,u},X_{flat,v})}{std(X_{flat,u})std(X_{flat,v})+\varepsilon }$$

where *u* and *v* denote distinct EEG channels, Cov(·) denotes the covariance calculation, and std(·) denotes the standard deviation. A small constant ε is added to ensure numerical stability. Learnable projections map each PCC row to query Q and key K vectors (Equation 16):

(16) $$Q=M_{PCC}W_{q}^{T},K=M_{PCC}W_{k}^{T}$$

The channel-mixing matrix is (Equation 17):

(17) $$A_{conn}=Softmax(\frac{QK^{T}}{\sqrt{d_{k}}})$$

The attention matrix left-multiplies the channel features (Equation 18):

(18) $$X_{conn}=A_{conn}X_{flat}$$

Following this enhancement, PCC is recomputed from $X_{conn}$ (Equation 19):

(19) $$M_{conn}=PCC(X_{conn})$$

A singleton feature-map dimension is added before convolution. Three Conv2D, batch-normalization, ReLU, and max-pooling blocks are then applied (Equation 20):

(20) $$\begin{array}{c} F_{c}=MaxPool(ReLU(BN(Conv2D(F_{c-1}))))\\ c=1,\;2,\;3 \end{array}$$

where $F_{0}=Unsqueeze(M_{conn})$. Two-dimensional global average pooling produces (Equation 21):

(21) $$F_{connect}=Flatten(GAP_{2d}(F_{3}))\in {\mathbb{R}}^{B\times 128}$$

#### 2.3.4 Feature fusion and classification

The two branch features are concatenated (Equation 22):

(22) $$F_{fusion}=[F_{temp};F_{connect}]$$

where [· ; ·] denotes the concatenation operation, yielding a unified feature vector $F_{fusion}\in \;{\mathbb{R}}^{B\times 256}$. Subsequently, the fused feature is fed into a fully connected classification layer (Equation 23):

(23) $$H=ReLU(FC(F_{fusion})),\;\;Z=FC(H)$$

where FC(·) represents a fully connected linear transformation, and *Z* denotes the class-logit matrix.

The class probabilities are obtained by applying the softmax function to the logits (Equation 24):

(24) $$T_{i,k}=\frac{exp(Z_{i,k})}{\sum_{j=1}^{K}exp(Z_{i,j})}$$

where $T_{i,\;\;k}$ is the predicted probability that the i-th sample belongs to the k-th class. The predicted class is given by (Equation 25):

(25) $${\hat{Y}}_{i}=argmax_{k}T_{i,k}$$

where ${\hat{Y}}_{i}$ denotes the predicted class label of the i-th sample.

The proposed DB-MDAFNet is optimized by minimizing the cross-entropy loss (Equation 26).

(26) $$L=-\frac{1}{B}\sum_{i=1}^{B}\sum_{j=1}^{K}Y_{i,j}\cdot log(T_{i,j})$$

where B denotes the batch size, K denotes the number of target classes, $Y_{i,j}$ is the ground-truth label of the i-th sample for the j-th class, and $T_{i,j}$ is the corresponding predicted probability. All trainable parameters are optimized end-to-end by backpropagation.

#### 2.3.5 Algorithm

The forward classification procedure of the DB-MDAFNet algorithm is presented in **Algorithm 1**.

Algorithm 1

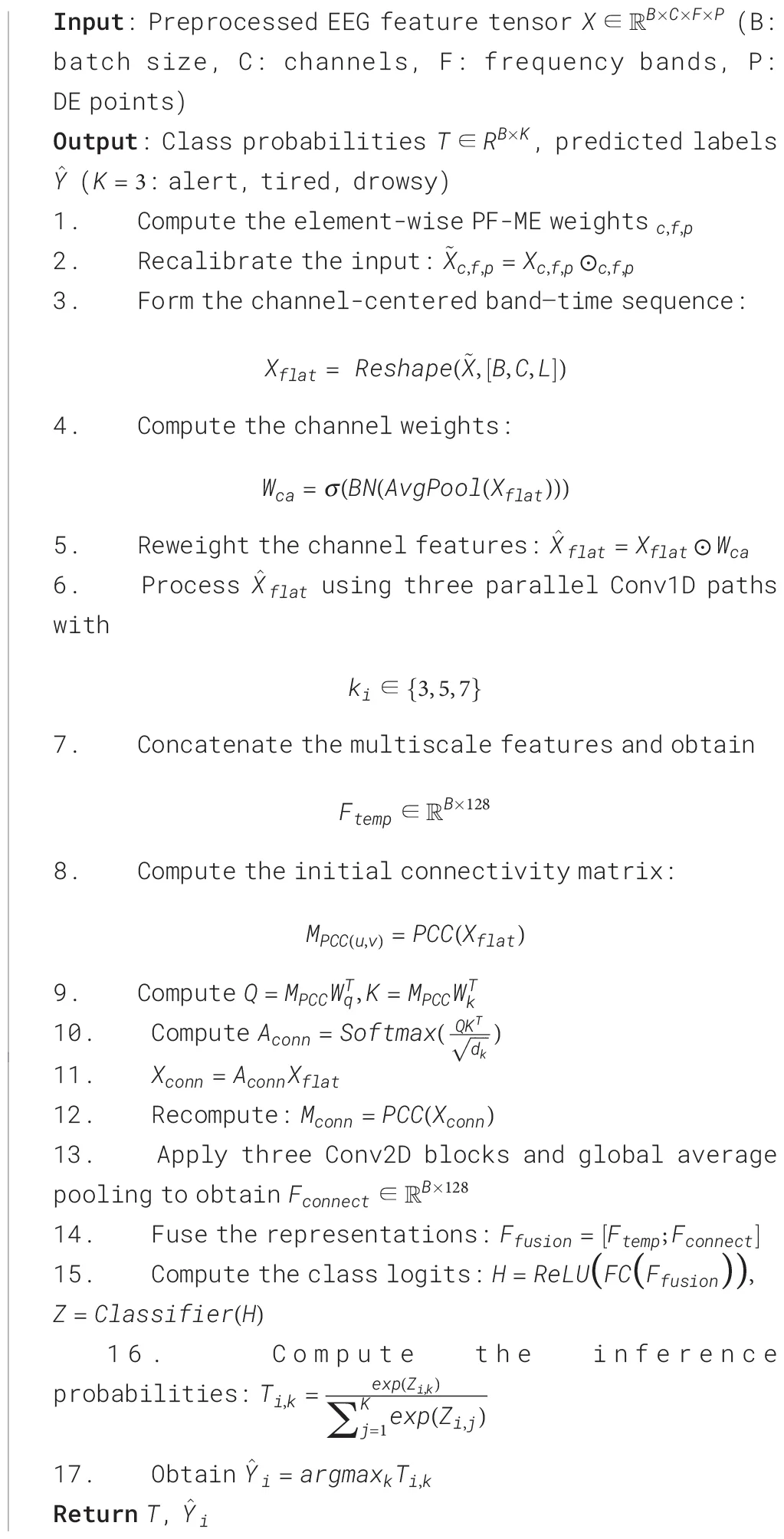

### 2.4 Experimental implementation details and evaluation metrics

DB-MDAFNet was implemented using PyTorch 2.1.0 (Paszke et al., 2019) and trained on an NVIDIA GeForce RTX 2060 GPU. The model was optimized using Adam (Kingma and Ba, 2015) with a batch size of 32 and an initial learning rate of 0.001. Batch normalization and dropout of 0.2 were applied to improve generalization performance and mitigate overfitting. The convolution kernel stride is 1.

A subject-dependent protocol was adopted for experimental validation. For both the SEED-VIG and DVG datasets, each subject’s data were partitioned into an 80% training allocation and a 20% independent testing set. This scheme ensured that the model was validated on entirely unseen data for each subject.

To ensure a fair comparison, all baseline models were independently retrained using the same preprocessed EEG data, input features, subject-specific data partitions, and evaluation protocol as the proposed model. Unless otherwise required by the original model architecture, the optimizer, learning rate, batch size, and predefined number of training epochs were kept consistent across models.

To rigorously evaluate the classifier’s performance, four core metrics were employed, including accuracy (Acc), macro-precision (Pre), macro-recall (Rec), and macro-F1-score (F1). These evaluation metrics were calculated based on the following equations (Equations 27–30):

(27) $$Acc=\frac{\sum_{k=1}^{K}TP_{k}}{N}$$

(28) $$Pre=\frac{1}{K}\sum_{k=1}^{K}\frac{TP_{k}}{TP_{k}+FP_{k}}$$

(29) $$Rec=\frac{1}{K}\sum_{k=1}^{K}\frac{TP_{k}}{TP_{k}+FN_{k}}$$

(30) $$F1=\frac{1}{K}\sum_{k=1}^{K}\frac{2\times Pre_{k}\times Rec_{k}}{Pre_{k}+Rec_{k}}$$

where *K* denotes the number of classes, and *N *denotes the total number of samples. $TP_{k}$, $FP_{k}$, and $FN_{k}$ represent true positives, false positives, and false negatives for class k, respectively.

## 3 Results

### 3.1 Within-subject experimental results

**Table 1** presents the average subject-dependent performance on the SEED-VIG and DVG datasets. In the current experiments, DB-MDAFNet achieved a mean classification accuracy of 85.58% and an F1 of 74.50% on the SEED-VIG dataset. Mean accuracy and F1 exceeded 97% for the DVG datasets (DVG1–DVG3).

**Table 1:** Subject-dependent classification performance (mean ± SEM).

Dataset		Acc (%)	Pre (%)	Rec (%)	F1 (%)
SEED-VIG		85.58 ± 1.64	80.98 ± 2.81	73.08 ± 2.34	74.50 ± 2.49
DVG	DVG1	98.24 ± 0.62	98.40 ± 0.56	98.25 ± 0.61	98.22 ± 0.63
	DVG2	97.12 ± 1.17	97.47 ± 1.00	97.10 ± 1.18	97.06 ± 1.20
	DVG3	98.56 ± 0.61	98.70 ± 0.53	98.54 ± 0.62	98.54 ± 0.31

### 3.2 Comparative experiments

DB-MDAFNet was compared with some representative EEG classification models. These models included HMS-TENet (Tang et al., 2024), T-A-MFFNet (Peng et al., 2023), M-ShallowConvNet (Kim et al., 2022), TSception (Ding et al., 2023), LGGNet (Ding et al., 2024), E-5-D-BCDRNet (Li et al., 2023), and SFT-Net (Gao et al., 2024). Comparisons were performed on SEED-VIG and DVG2 using accuracy, precision, recall, and F1. To assess whether these improvements were statistically significant, two-sided paired Wilcoxon signed-rank tests were used to compare participant-level accuracies (SEED-VIG: n = 23; DVG2: n = 12). The unit of analysis was the individual participant. For each comparison, the subject-level classification accuracy of the proposed model was paired with that of the corresponding baseline model.

As shown in **Table 2**, DB-MDAFNet obtained the highest mean value for each reported metric on the SEED-VIG dataset. Its accuracy and F1 were 85.58% and 74.50%, exceeding those of the strongest baseline LGGNet by 1.40% and 3.21%, respectively.

**Table 2:** Comparative experimental results of the SEED-VIG dataset (mean ± SEM).

Model	Acc (%)	Pre (%)	Rec (%)	F1 (%)	*p*-value (Acc)	Significant
HMS-TENet (Tang et al., 2024)	79.37 ± 1.99	65.24 ± 2.57	62.46 ± 2.58	62.04 ± 2.74	<0.0001	√
T-A-MFFNet (Peng et al., 2023)	78.90 ± 2.11	60.94 ± 3.41	57.12 ± 3.02	55.23 ± 3.31	<0.0001	√
M-ShallowConvNet (Kim et al., 2022)	82.83 ± 1.78	72.79 ± 2.86	67.93 ± 2.25	67.37 ± 2.43	<0.0001	√
TSception (Ding et al., 2023)	83.64 ± 1.89	73.39 ± 3.33	68.82 ± 2.77	68.85 ± 2.88	0.0124	√
LGGNet (Ding et al., 2024)	84.18 ± 1.79	77.49 ± 2.63	70.79 ± 2.74	71.29 ± 2.74	0.0112	√
DB-MDAFNet	85.58 ± 1.64	80.98 ± 2.81	73.08 ± 2.34	74.50 ± 2.49	-	-

On the DVG2 dataset (**Table 3**), DB-MDAFNet surpassed all baselines with an accuracy of 97.12%, and Pre, Rec, and F1 reached 97.47%, 97.10%, and 97.06%, respectively. Relative to M-ShallowConvNet, these results represented gains of 2.41% in accuracy and 2.34% in F1.

**Table 3:** Comparative experimental results of the self-constructed dataset (DVG2, mean ± SEM).

Model	Acc (%)	Pre (%)	Rec (%)	F1 (%)	*p*-value (Acc)	Significant
E-5-D-BCDRNet (Li et al., 2023)	79.17 ± 3.60	80.16 ± 3.51	79.18 ± 3.59	78.23 ± 3.98	<0.001	√
M-ShallowConvNet (Kim et al., 2022)	94.71 ± 1.67	95.23 ± 1.54	94.72 ± 1.67	94.72 ± 1.67	0.0313	√
T-A-MFFNet (Peng et al., 2023)	81.41 ± 3.00	83.52 ± 2.91	81.54 ± 3.04	80.59 ± 3.48	<0.001	√
SFT-Net (Gao et al., 2024)	83.17 ± 2.38	86.31 ± 1.78	83.10 ± 2.37	82.33 ± 2.64	<0.001	√
LGGNet (Ding et al., 2024)	92.63 ± 2.04	92.83 ± 2.02	92.66 ± 2.03	92.56 ± 2.04	0.0039	√
DB-MDAFNet	97.12 ± 1.17	97.47 ± 1.00	97.10 ± 1.18	97.06 ± 1.20	-	-

### 3.3 Ablation study

To quantify the contribution of each core component within DB-MDAFNet, a systematic ablation study was conducted using a within-subject design. We evaluated four model variants: (1) the full DB-MDAFNet, (2) without the PF-ME module, (3) without the CA-MT module, and (4) without the FC-TE module.

As shown in **Table 4**, the full DB-MDAFNet achieved an accuracy of 98.24% and an F1 of 98.22%. Removing PF-ME reduced accuracy by 0.53% and F1 by 0.55%. Removing the CA-MT and FC-TE modules reduced accuracy to 77.17% and 81.09%, respectively. These reductions indicate that both the CA-MT and the FC-TE are essential components of the architecture.

**Table 4:** Ablation results on DVG1.

Dataset	Acc (%)	Pre (%)	Rec (%)	F1 (%)
Full	98.24	98.40	98.25	98.22
w/o PF-ME	97.71	97.70	97.81	97.67
w/o CA-MT	77.17	76.30	76.34	73.17
w/o FC-TE	81.09	82.17	81.21	80.55

### 3.4 Computational complexity

The computational complexity of DB-MDAFNet was evaluated under the DVG input configuration and compared with four representative baseline models. As shown in **Table 5**, DB-MDAFNet contained 0.185 M trainable parameters, required approximately 0.044 G FLOPs per sample, and occupied 0.704 MiB. It had substantially fewer parameters and a smaller model size than T-A-MFFNet and E-5-D-BCDRNet, while requiring fewer FLOPs than E-5-D-BCDRNet and SFT-Net. Although SFT-Net and LGGNet contained fewer parameters, and T-A-MFFNet and LGGNet required slightly fewer FLOPs, DB-MDAFNet maintained fewer than 0.2 M parameters, less than 0.05 G FLOPs, and a model size below 1 MB. Taken together with the classification results, these results suggest that DB-MDAFNet achieves a favorable trade-off among classification performance, model compactness, and computational cost.

**Table 5:** Computational complexity comparison of DB-MDAFNet and representative baseline models under the DVG input configuration.

Dataset	Model	Params (M)	FLOPs (G)	Model size (MiB)
DVG	DB-MDAFNet	0.185	0.044	0.704
	T-A-MFFNet (Peng et al., 2023)	0.909	0.041	3.467
	E-5-D-BCDRNet (Li et al., 2023)	1.199	24.139	4.575
	SFT-Net (Gao et al., 2024)	0.141	0.085	0.537
	LGGNet (Ding et al., 2024)	0.102	0.041	0.390

### 3.5 Model visualization and interpretability

**Figure 4:**
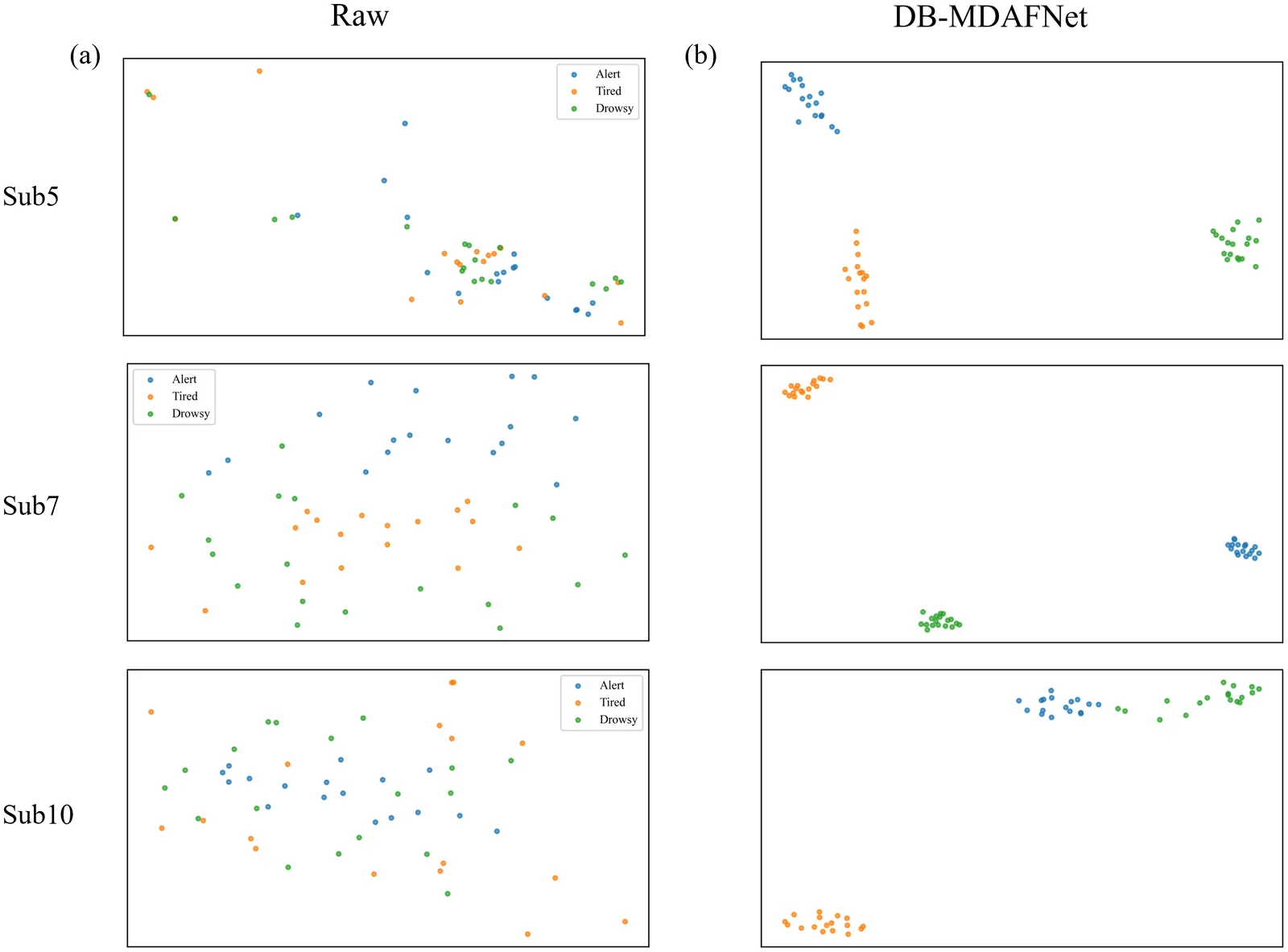
Two-dimensional t-SNE visualization of raw and fused features for sub5, sub7, and sub10. **(a)** Raw DE features; **(b)** fused DB-MDAFNet features. Colors denote alert, tired, and drowsy samples.

To examine the discriminative structure and internal feature-focus patterns learned by DB-MDAFNet, we visualized the fused feature representations and the channel-related weights generated by the two branches. **Figure 4** illustrates the evolution of feature manifolds from raw DE features to fused DB-MDAFNet features across randomly selected subjects (sub5, sub7, and sub10) of DVG1, colored by the three states (alert, tired, and drowsy). As demonstrated in **Figure 4a**, the raw DE features exhibit a highly mixed distribution for all visualized subjects. After processing by DB-MDAFNet, the class overlap was reduced, yielding compact and well-separated clusters (**Figure 4b**).

**Figure 5:**
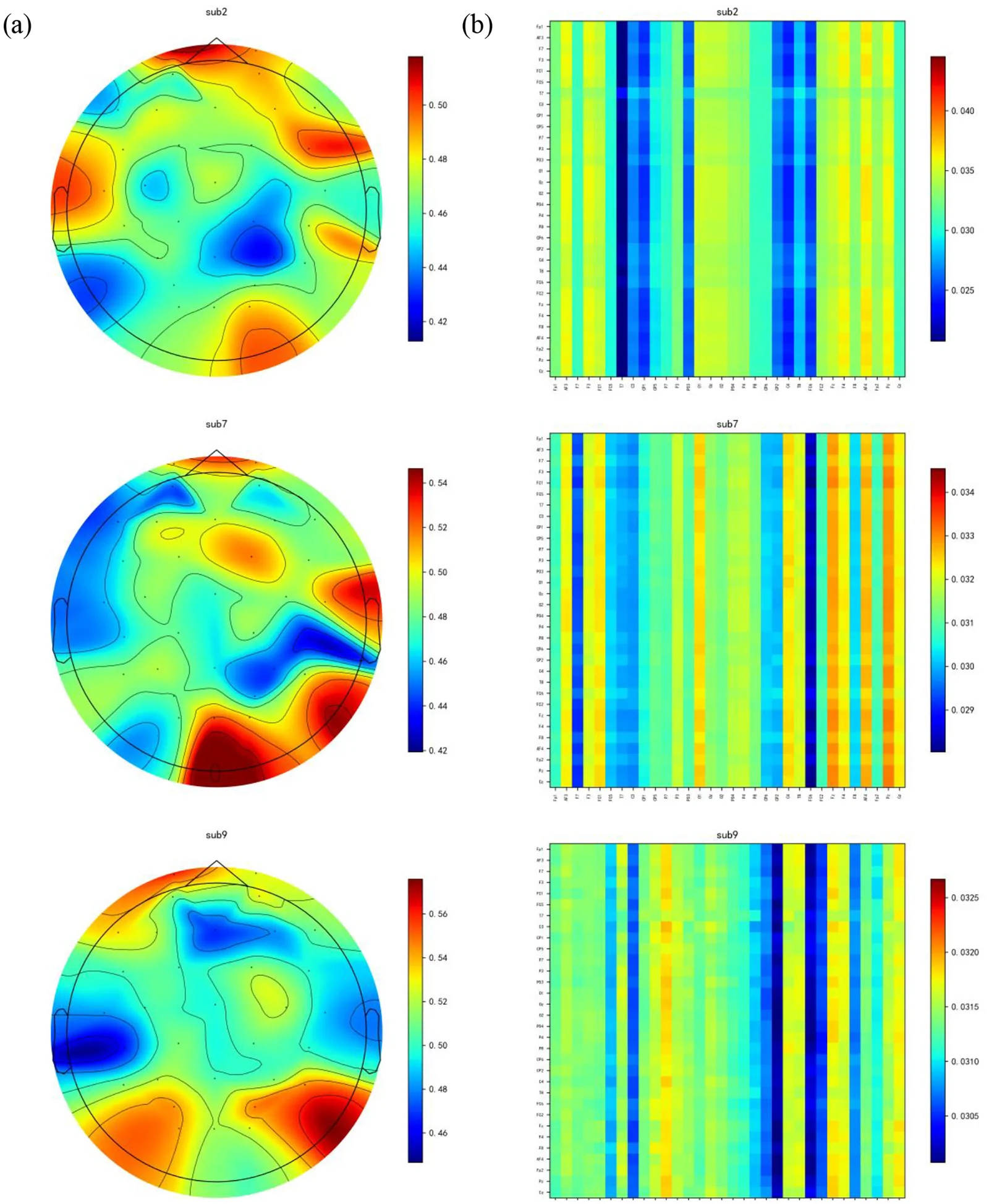
Channel-weight and channel-attention visualization of DB-MDAFNet for subjects (sub2, sub7, and sub9). **(a)** Scalp topographic map of attention weights learned by the CA-MT module; **(b)** FC-TE channel-attention matrices, with destination/query channels on the vertical axis and source/key channels on the horizontal axis.

To examine the internal spatial focus of the model, we visualized the attention weights from the CA-MT module and mapped them onto the scalp topography. **Figure 5a** illustrates the weights for three randomly selected subjects from the DVG1 dataset (sub2, sub7, and sub9). For these participants, relatively high weights appeared over frontal, parietal, and occipital electrodes. These weights indicate the relative contribution of the corresponding channel features to the model’s classification process.

**Figure 5b** presents the subject-specific PCC-guided channel-attention matrices generated by the FC-TE branch. Each element represents the normalized weight assigned to an input channel when updating the representation of a query channel. Several columns showed relatively high weights across multiple query channels, indicating that the corresponding input channels contributed more strongly to feature aggregation. These high-weight channels were mainly located over frontal and occipital scalp areas and partially overlapped with the channels receiving relatively high weights in the CA-MT branch. Importantly, these matrices represent learned channel-mixing patterns.

## 4 Discussion

The experimental results show that DB-MDAFNet achieves competitive performance in subject-dependent EEG-based fatigue classification. By jointly modeling spectral-temporal dynamics and functional connectivity patterns, the model provides a more comprehensive representation of fatigue-related EEG changes than methods relying on a single feature. The performance degradation observed after removing either the CA-MT or the FC-TE branch further supports the effectiveness of combining these two computational representations within the proposed classification framework.

The performance of DB-MDAFNet may be attributed to its joint use of band-specific temporal features and statistical relationships among EEG channels. Previous studies have reported that fatigue-related EEG changes involve both frequency-specific changes in neural oscillations and altered functional coordination among cortical regions (Li et al., 2020; Qi et al., 2020; Tran et al., 2020). Motivated by these findings, the CA-MT branch focuses on local spectral-temporal dynamics through multi-scale one-dimensional convolutions and channel attention, thereby capturing short-term rhythm fluctuations and band-specific variations during fatigue transitions. In parallel, the FC-TE branch models inter-channel dependencies using PCC-based inter-channel connectivity representations, which describe network-level coordination across brain regions. By integrating local oscillatory features with global connectivity information, DB-MDAFNet provides a more comprehensive representation of fatigue-related neural activity and enhances the discrimination of different fatigue states.

The PF-ME module further improves feature representation by introducing lightweight feature recalibration. Based on the parameter-free SimAM mechanism (Yang et al., 2021), PF-ME assigns adaptive importance weights to channel-frequency-temporal feature units without introducing additional trainable parameters. This design enables the model to emphasize features that are more relevant to fatigue detection while preserving a compact architecture. PF-ME serves as a feature-refinement module that enhances fatigue-related representations with limited computational overhead.

The visualization analyses provide descriptive insight into the internal representations and feature-focus patterns learned by DB-MDAFNet. The channel-weight topographic maps learned by the CA-MT module show relatively higher weights over the prefrontal, parietal, and occipital areas. This spatial attention pattern is consistent with prior neurophysiological evidence, as prefrontal and parietal areas are commonly implicated in executive control and sustained attention (Corbetta and Shulman, 2002), whereas occipital areas are primarily involved in visual information processing (Zeki et al., 1991) during vigilance-related tasks. The FC-TE channel-attention matrices further reveal patterns of inter-channel dependencies. Notably, stronger attention weights are observed over frontal, parietal, and occipital regions, suggesting that DB-MDAFNet may be sensitive to coordinated interactions among fatigue-related cortical areas. Interactions involving frontal and parietal channels may be related to attentional control and executive regulation, and interactions involving occipital channels may reflect the contribution of visual information processing during vigilance-related tasks. Importantly, the overlap between high-weight regions in the CA-MT topographic maps and FC-TE channel-attention matrices indicates that the two branches capture fatigue-related patterns from complementary perspectives. CA-MT emphasizes region-specific spectral-temporal activity, whereas FC-TE highlights connectivity-related inter-channel interactions. Taken together, these visualization results suggest that DB-MDAFNet may learn physiologically meaningful representations from both intra-channel and inter-channel interactions.

Several limitations should be considered. The current experiments adopted a subject-dependent evaluation protocol. Although the results demonstrate the ability of DB-MDAFNet to distinguish fatigue stages within individual participants, its performance under cross-session and cross-dataset conditions remains unknown. Future studies should therefore evaluate the model using leave-one-subject-out validation, independent external datasets, and domain-generalization or adaptation strategies. Despite its compact architecture, DB-MDAFNet requires further validation for real-time fatigue monitoring under online EEG acquisition, and artifact-contaminated recording conditions. Finally, larger-scale datasets involving more diverse subjects, multimodal physiological signals, and fatigue-inducing scenarios are needed to further assess the generalizability and ecological validity of the proposed framework.

Overall, DB-MDAFNet provides an effective and interpretable framework for subject-dependent EEG-based fatigue classification. By integrating multi-scale spectral-temporal modeling, PCC-derived inter-channel relation encoding, and lightweight multidimensional feature recalibration, the model characterizes complementary fatigue-related EEG patterns from both local and network-level perspectives. While further validation is still required in more challenging generalization and real-world application settings, the proposed dual-branch design offers a promising direction for EEG-based fatigue monitoring. It may provide useful methodological insights for broader cognitive state recognition tasks.

## 5 Conclusion

This study introduced DB-MDAFNet, a dual-branch framework that integrates channel-aware band-time features with PCC-derived inter-channel representations for EEG fatigue classification. Under subject-dependent evaluation, DB-MDAFNet achieved the highest mean performance among the evaluated methods on SEED-VIG and DVG2 and maintained strong performance across DVG1–DVG3. The ablation results indicated that PF-ME, CA-MT, and FC-TE each contributed to model performance. Visualization analyses suggested that the learned representations contained class-discriminative representations and spatially structured channel-weighting patterns. The proposed model provides a lightweight and interpretable architecture for EEG-based fatigue monitoring.

## Data Availability

The raw data supporting the conclusions of this article will be made available by the authors, without undue reservation.
